# Brugada Phenocopy Associated With the Tyrosine Kinase Inhibitor Avapritinib During Febrile Illness

**DOI:** 10.1016/j.jaccas.2026.107364

**Published:** 2026-03-16

**Authors:** Obaid I. Haque, Obaid ur Rehman, Leen Othman, Mansoor Mozayan, Konstantinos N. Aronis

**Affiliations:** a Internal Medicine, MedStar Health Georgetown University, Baltimore, Maryland, USA; b Cardiology at MedStar Franklin Square Medical Center, Baltimore, Maryland, USA; c Division of Cardiology, Johns Hopkins School of Medicine, Johns Hopkins Hospital, Baltimore, Maryland, USA

**Keywords:** avapritinib, case report, channelopathy, Brugada pattern, Brugada syndrome, tyrosine kinase inhibitor

## Abstract

**Background:**

Brugada phenocopy is a reversible Brugada pattern on an electrocardiogram (ECG) caused by identifiable clinical conditions (such as fever, electrolyte abnormalities, or drug exposure) in patients without true congenital Brugada syndrome, which is an inherited arrhythmogenic disorder often associated with *SCN5A* mutations.

**Case Summary:**

A 53-year-old man with metastatic myeloid sarcoma presented with high-grade fever and chest pain. He was previously treated with imatinib, discontinued because of severe skin rash, followed by dasatinib, and most recently avapritinib, initiated 3 weeks before presentation. The initial 12-lead ECG demonstrated a spontaneous type 1 Brugada pattern in leads V_1_ and V_2_. Avapritinib was withheld, and Brugada ECG pattern resolved with defervescence. Genetic testing identified a heterozygous missense variant in the *JPH2* gene.

**Discussion:**

We report a case of type 1 Brugada ECG pattern unmasked by febrile illness after avapritinib initiation, with an incidental finding of heterozygous missense variant in the *JPH2* gene.

**Take-Home Message:**

This case introduces a previously unrecognized association between avapritinib and Brugada pattern and suggests avapritinib as a potential pharmacologic trigger in susceptible individuals.

## Visual Summary

**Figure:**
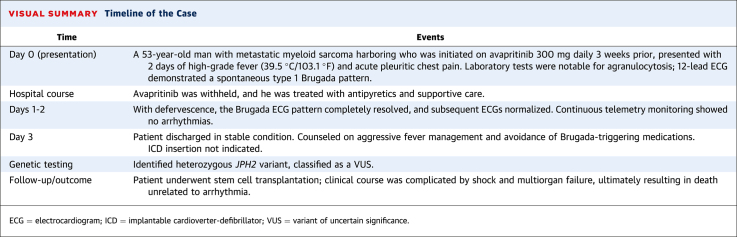

## History of Presentation

A 53-year-old man with metastatic myeloid sarcoma presented to the emergency department with fever (self-recorded maximum temperature: 107 °F [41.7 °C]) for 2 days, and severe central chest pain for a few hours, pleuritic in nature, aggravated by lying flat and relieved by sitting upright. He denied any palpitations, dizziness, light-headedness, presyncope, or syncope. He had no prior personal or family history of syncope, seizures, sudden cardiac death, or known arrhythmias. He denied any history of smoking, alcohol, or illicit substance use. Vital signs were notable for a body temperature of 103.1 °F (39.5 °C) and heart rate of 110 beats/min. He appeared cachectic and weak, with an otherwise unremarkable physical examination.

**Take-Home Messages**

• Avapritinib, in the presence of fever, was temporally associated with a reversible Brugada ECG pattern, representing a previously unreported potential cardiac toxicity warranting further pharmacovigilance.
• Asymptomatic patients with drug-induced Brugada pattern have a low risk of arrhythmias; and management involves withdrawal of the offending agent, aggressive fever control, and close clinical monitoring.

## Past Medical History

The patient initially presented with left arm pain 1 year prior to the current presentation. Sequential imaging revealed marrow signal abnormalities in the left humerus with soft tissue extension. Complete blood count showed eosinophilia (1.05 × 10^9^/L). Muscle biopsy demonstrated aggressive malignant neoplasm infiltrating skeletal muscle. Positron emission tomography/computed tomography showed diffuse FDG (^18^F-fluorodeoxyglucose)–avid marrow disease, particularly in the left proximal humerus and bilateral femoral regions, with FDG-avid left supraclavicular lymphadenopathy. Bone marrow biopsy revealed hypercellular marrow (>90% cellularity) with marked myeloid hyperplasia, 4.5% myeloblasts, and increased reticulin fibrosis (MF 2-3). Cytogenetics demonstrated trisomy 8. FISH (fluorescence in situ hybridization) was positive for *PDGFRA* rearrangement (*FIP1L1::PDGFRA*), GOF *FGFR1*/trisomy 8, and gain of *MYC*. Next-generation sequencing panel (CARIS) identified *BCOR* pathogenic variant, PDL1-negative, microsatellite stable, low tumor mutational burden (4 mutations/Mb), and low loss of heterozygosity (7%). A biopsy of the left upper extremity humerus soft tissue mass confirmed myeloid sarcoma. Imatinib was initiated and achieved a molecular response, but it was discontinued given persistent, treatment-limiting skin rash.

Within weeks after the interruption, the patient developed new-onset dysphagia and back pain; imaging revealed extensive cervical and retroperitoneal lymphadenopathy, consistent with widespread metastatic involvement. A core biopsy of a supraclavicular lymph node verified active myeloid disease. He received gemtuzumab ozogamicin for debulking, along with dexamethasone, which was switched to dasatinib. Subsequent imaging revealed persistent disease, prompting a switch to nilotinib and initiation of salvage high-dose cytarabine (HiDAC) 3 months before the current presentation. His post-HiDAC course was complicated by refractory musculoskeletal pain and persistent fevers without an identifiable source, necessitating discontinuation of nilotinib and transition to avapritinib 300 mg daily 3 weeks before the current presentation.

## Investigations

**Figure 1:**
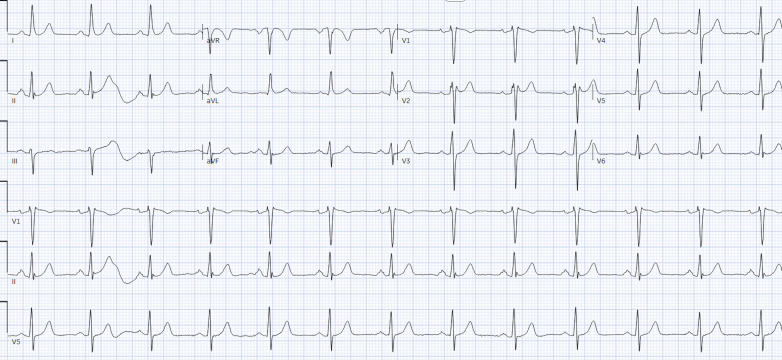
Baseline 12-Lead Electrocardiogram Before Initiation of Avapritinib Demonstrating Normal Sinus Rhythm With No ST-Segment Abnormalities in the Right Precordial Leads (V_1_ and V_2_) Standard lead placement at the fourth intercostal space.

**Figure 2:**
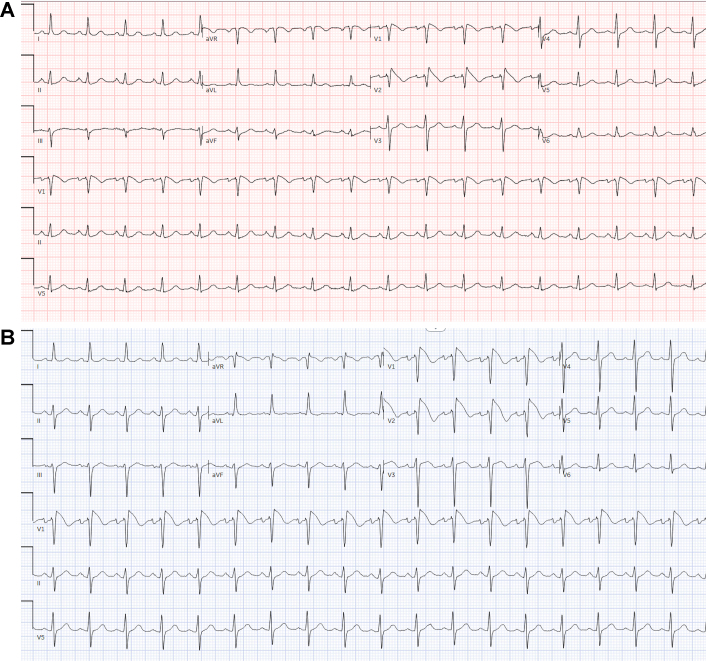
Electrocardiogram on Presentation While on Avapritinib, Repeat Electrocardiogram With High Chest Leads (A) A 12-lead electrocardiogram obtained during febrile presentation (39.5 °C) while on avapritinib demonstrated a spontaneous type 1 Brugada pattern with coved ST-segment elevation ≥2 mm followed by negative T waves in leads V_1_ and V_2_. Standard lead placement at the fourth intercostal space. (B) A 12-lead electrocardiogram with high precordial lead placement (V_1_ and V_2_ positioned at the second intercostal space) confirmed the type 1 Brugada pattern with coved ST-segment elevation and T-wave inversion in the right precordial leads. High lead placement increases diagnostic sensitivity for Brugada pattern by approximately 1.5-fold compared with standard positioning.

Initial laboratory studies demonstrated agranulocytosis (absolute neutrophil count: 0 cells/μL) and mildly elevated high-sensitivity troponin I (39 ng/L), which normalized within 24 hours. Blood cultures were obtained and remained negative. The laboratory results are summarized in Table 1. The patient's baseline electrocardiogram (ECG) performed before initiation of chemotherapy was normal (Figure 1). The initial 12-lead ECG on presentation demonstrated coved ST-segment elevation >2 mm in leads V_1_ and V_2_ followed by negative T waves (Figure 2A), consistent with type 1 Brugada pattern. Repeat ECG with high chest leads also revealed type 1 Brugada pattern (Figure 2B). Coronary angiogram showed no obstructive coronary disease. Transthoracic echocardiography revealed normal biventricular size and systolic function with a left ventricular ejection fraction of 60%, without any structural or valvular abnormalities. Continuous telemetry over the next 48 hours showed no arrhythmias.

**Table 1:** Initial Laboratory Results

Parameter	Result	Reference Range
High-sensitivity troponin I (ng/L)	39 → 15	0-30
NT-proBNP (pg/mL)	264.0	≤900
Serum creatinine (mg/dL)	1.01	0.6-1.30
Lactate (mmol/L)	1.7	0.5-2.0
Sodium (mmol/L)	137	10-49
Potassium (mmol/L)	4.8	3.5-5.1
Magnesium (mg/dL)	1.7	1.6-2.4
WBC count (k/μL)	0.43	4.5-11.0
Absolute neutrophil count (k/μL)	0.0	1.5-7.5
Differential WBC count		
Neutrophils	0%	31%-76%
Lymphocyte	80%	24%-44%
Monocytes	10%	3%-12%
Atypical lymphocytes	10%	—
Hemoglobin (g/dL)	10.1	13.9-16.3

NT-proBNP = N-terminal pro–B-type natriuretic peptide; WBC = white blood cell.

## Differential Diagnosis

The initial presentation raised concern for ST-segment elevation myocardial infarction; however coronary angiography was unremarkable. Myocarditis was considered, however the rapid normalization of troponin and absence of arrhythmias made myocarditis less likely, and cardiac magnetic resonance imaging was not pursued.

## Management

Avapritinib was withheld, and he was treated with acetaminophen and supportive care. After defervescence, a repeat ECG demonstrated the resolution of the type 1 Brugada pattern. He was discharged in stable condition with close cardiology and oncology follow-up and was counseled on the importance of early and aggressive antipyretic treatment for any future febrile illnesses, along with avoidance of potential triggers (large meals, alcohol, stimulants, energy drinks, or illicit drugs). Reinitiation of avapritinib was considered; however the decision was made to proceed with stem cell transplantation as a definitive treatment. Family members were advised regarding genetic screening.

## Outcome and Follow-Up

Genetic testing identified a heterozygous missense variant in the *JPH2* gene (NM_020433.4:c.50G>C, p.Gly17Ala). This variant, located in exon 1, alters a conserved glycine residue and is not observed at a significant frequency in large population databases, including gnomAD (Genome Aggregation Database). In silico predictive modeling suggests a deleterious impact on the structure and/or function of the junctophilin-2 protein; it is classified as a variant of uncertain significance (VUS).^1^

The patient was followed up in the oncology clinic and was initiated on a reduced-intensity conditioning regimen in preparation for a non-myeloablative haploidentical peripheral blood stem cell transplant (PBSCT) from his son, who was an ABO major-incompatible donor (A+ to B+). Unfortunately, soon after transplantation, he developed neutropenic fever and tested positive for a non–COVID-19 coronavirus and was admitted to the critical care unit. His clinical course rapidly deteriorated, with the onset of acute hypoxic respiratory failure requiring mechanical ventilation. He subsequently developed multiorgan failure and, despite aggressive supportive measures, succumbed to his illness.

## Discussion

This report illustrates a case of type 1 Brugada ECG pattern in a middle-aged male patient with metastatic myeloid sarcoma shortly after the initiation of avapritinib, a selective tyrosine kinase inhibitor (TKI) targeting mutant forms of *KIT* and *PDGFRA*. Brugada pattern refers to the characteristic ECG findings in the absence of clinical symptoms or family history of sudden cardiac death, whereas Brugada syndrome (BrS) is diagnosed when these ECG findings are present in conjunction with clinical features such as unexplained syncope, documented ventricular arrhythmias, or resuscitated cardiac arrest, after exclusion of phenocopies.^2^ Around 25% of BrS cases are associated with loss-of-function mutation in the cardiac voltage-gated sodium channel gene *SCN5A*.^3^ Brugada ECG pattern is well known to be precipitated by environmental factors such as fever and noncardiac medications.^4^^,^^5^ However, there is no robust evidence that fever alone, without any underlying genetic abnormality or susceptibility, can cause a true Brugada pattern.^5^

In our literature review, we identified 3 reported cases of BrS associated with TKIs, including entrectinib and dasatinib (Table 2); to our knowledge, avapritinib has not previously been implicated.^6^, ^7^, ^8^ Unlike other TKIs such as nilotinib, vandetanib, sunitinib, and dasatinib, which have been extensively characterized for their effects on hERG (human ether-a-go-go–related gene), late sodium current (INa-L), and L-type calcium current (ICaL), avapritinib does not appear in published ion channel screening studies or the CiPA (Comprehensive in vitro Proarrhythmia Assay) database.^9^ Preclinical studies have demonstrated that avapritinib inhibits the hERG potassium channel with an IC_50_ of 2.4 μM, which is approximately 1.5 times the unbound maximum plasma concentration achieved at the therapeutic 300-mg dose in humans.^10^ However, these evaluations focused exclusively on repolarization abnormalities mediated by potassium channel inhibition and did not characterize potential effects on cardiac sodium channels. This is a critical gap because drug-induced BrS is most commonly unmasked by agents that affect ventricular sodium currents through inhibition of Nav1.5 encoded by *SCN5A*, rather than through potassium channel blockade.

**Table 2:** Previously Reported Cases on TKIs and Brugada Syndrome

First Author (Year)	Age/Sex	Malignancy	Drug	Onset	Symptom	Genetic Testing	Management
Sgherza et al (2013)^6^	69/Male	Chronic myeloid leukemia	Dasatinib	1 y	Syncope	Not done	TKI continued after ICD insertion
Nardin et al (2017)^7^	50/Male	Melanoma	Dabrafenib and trametinib	5 mo	Asymptomatic	Not done	TKI discontinued
Futamura et al (2023)^8^	81/Male	*ROS1*-positive NSCLC	Entrectinib	3 d	Chest pain	Not done	TKI discontinued
Present case (2026)	53/Male	Myeloid sarcoma	Avapritinib	3 wk	Chest pain	*JPH2* (VUS)	TKI discontinued for stem cell transplant

ICD = implantable cardioverter-defibrillator; NSCLC = non–small cell lung cancer; TKI = tyrosine kinase inhibitor; VUS = variant of uncertain significance.

Genetic testing identified a novel heterozygous missense variant in the *JPH2* gene: NM_020433.4:c.50G>C, resulting in a predicted amino acid substitution p.(Gly17Ala). *JPH2* encodes junctophilin-2, a critical component of the junctional membrane complex predominantly expressed in cardiac myocytes, where it contributes to excitation-contraction coupling.^11^ This has been associated with other structural and conduction phenotypes (including hypertrophic cardiomyopathy, dilated cardiomyopathy, atrial fibrillation, cardiac conduction system disease, and cases of sudden cardiac death); current evidence does not support asserting a causal role for this specific *JPH2* VUS in Brugada pattern in our patient.^12^ Furthermore, there are no available family segregation data or definitive functional studies linking this specific *JPH2* variant to BrS, and as it is classified as a VUS without segregation or functional confirmation, it should be regarded as coincidental to the clinical presentation.

The distinction between Brugada phenocopy and BrS is critical for management and prognosis. Asymptomatic patients with drug-induced Brugada pattern are at low arrhythmic risk and may be managed conservatively, whereas patients with true BrS, particularly those with spontaneous type 1 ECG and symptoms, are at higher risk (2.3%-3.7%/year with cardiogenic syncope) and may require implantable cardioverter-defibrillator therapy.^2^ The management of drug-induced Brugada pattern follows established principles: discontinue the offending agent, aggressively treat fever with antipyretics, and monitor for arrhythmias. In our patient, discontinuation of avapritinib and antipyretic care led to rapid ECG normalization, with no observed arrhythmias during 48 hours of continuous monitoring. The patient had no history of cardiac arrest, ventricular arrhythmias, or syncope, and the Brugada pattern was attributable to reversible triggers (fever and possible avapritinib exposure); therefore, implantable cardioverter-defibrillator placement was not indicated. However, the diagnosis warrants future precaution. Patients should be counseled to avoid known precipitants, including large meals, excessive alcohol, stimulants (caffeine, energy drinks), and any medications listed on the Brugada “avoid” list (brugadadrugs.org). Although Food & Drug Administration labeling does not specify cardiac monitoring for avapritinib, a reasonable approach based on general TKI safety principles would include a baseline ECG, a repeat ECG 14 days after initiation, and additional ECGs as clinically indicated.^13^

## Conclusions

This case describes a reversible type 1 Brugada ECG pattern associated with fever and possible avapritinib exposure. While causality cannot be established from a single case, it suggests that newer TKIs may have unrecognized electrophysiologic effects, underscoring the importance of clinical vigilance and case reporting.

## Funding Support and Author Disclosures

The authors have reported that they have no relationships relevant to the contents of this paper to disclose.

**Visual Summary:** Timeline of the Case

Time	Events
Day 0 (presentation)	A 53-year-old man with metastatic myeloid sarcoma who was initiated on avapritinib 300 mg daily 3 weeks prior, presented with 2 days of high-grade fever (39.5 °C/103.1 °F) and acute pleuritic chest pain. Laboratory tests were notable for agranulocytosis; 12-lead ECG demonstrated a spontaneous type 1 Brugada pattern.
Hospital course	Avapritinib was withheld, and he was treated with antipyretics and supportive care.
Days 1-2	With defervescence, the Brugada ECG pattern resolved and subsequent ECGs normalized. Continuous telemetry monitoring revealed no arrhythmias.
Day 3	The patient was discharged in stable condition with counseling on prompt fever management and avoidance of Brugada-provoking triggers. Given the reversible nature of Brugada phenocopy, ICD implantation was not indicated.
Genetic testing	Identified heterozygous *JPH2* variant, classified as a VUS.
Follow-up/outcome	Patient underwent stem cell transplantation; clinical course was complicated by shock and multiorgan failure, ultimately resulting in death unrelated to arrhythmia.

ECG = electrocardiogram; ICD = implantable cardioverter-defibrillator; VUS = variant of uncertain significance.
